# Safety and Risk Factors of Needle Thoracentesis Decompression in Tension Pneumothorax in Patients over 75 Years Old

**DOI:** 10.1155/2023/2602988

**Published:** 2023-05-04

**Authors:** Yanhu Wang, Lei Wang, Cheng Chen, Yifan Que, Yinyi Li, Jiang Luo, Ming Yin, Miao Lv, Guogang Xu

**Affiliations:** ^1^The Second Medical Center and National Clinical Research Center for Geriatric Diseases, Chinese PLA General Hospital, Medical School of Chinese PLA, Beijing, China; ^2^Department of Obstetrics and Gynecology, The First Affiliated Hospital of Xi'an Jiaotong University, Xi'an, Shaanxi, China; ^3^Department of Emergency, The Second Medical Center and National Clinical Research Center for Geriatric Diseases, Chinese PLA General Hospital, Beijing, China; ^4^The Third Medical Center, Chinese PLA General Hospital, Medical School of Chinese PLA, Beijing, China

## Abstract

**Background:**

There are very few professional recommendations or guidelines on the needle thoracentesis decompression (NTD) for the tension pneumothorax in the elderly. This study aimed to investigate the safety and risk factors of tension pneumothorax NTD in patients over 75 years old based on CT evaluation of the chest wall thickness (CWT).

**Methods:**

The retrospective study was conducted among 136 in-patients over 75 years old. The CWT and closest depth to vital structure of the second intercostal space at the midclavicular line (second ICS-MCL) and the fifth intercostal space at the midaxillary line (fifth ICS-MAL) were compared as well as the expected failure rates and the incidence of severe complications of different needles. We also analyzed the influence of age, sex, presence or absence of chronic obstructive pulmonary disease (COPD), and body mass index (BMI) on CWT.

**Results:**

The CWT of the second ICS-MCL was smaller than the fifth ICS-MAL both on the left and the right side (*P* < 0.05). The success rate associated with a 7 cm needle was significantly higher than a 5 cm needle (*P* < 0.05), and the incidence of severe complications with a 7 cm needle was significantly less than an 8 cm needle (*P* < 0.05). The CWT of the second ICS-MCL was significantly correlated with age, sex, presence or absence of COPD, and BMI (*P* < 0.05), whereas the CWT of the fifth ICS-MAL was significantly correlated with sex and BMI (*P* < 0.05).

**Conclusion:**

The second ICS-MCL was recommended as the primary thoracentesis site and a 7 cm needle was advised as preferred needle length for the older patients. Factors such as age, sex, presence or absence of COPD, and BMI should be considered when choosing the appropriate needle length.

## 1. Introduction

The elderly patient population is rapidly growing due to improvements in preventive health service and medical care, which leads to increasing populations of elderly trauma patient. According to 2016 National Trauma Databank (NTDB), 31% of trauma patients were at least 65 years old [1]. Elderly trauma patients have worse outcomes compared with younger patients with similar injuries [2–5]. Therefore, how to improve treatment of trauma in elderly patients is an important research topic.

Tension pneumothorax is a fatal disease causing acute and severe compromise of patients' ventilation and circulation, in which case immediate decompression is necessary for better prognosis [6]. A previous study has shown that the incidence of tension pneumothorax varies from 0.2% to 1.7% in patients with prehospital trauma [7]. Several trauma guidelines [8–10] recommend needle thoracostomy as a life-saving intervention, with placement in the second intercostal space at the midclavicular line (second ICS-MCL), the fourth intercostal space at the anterior axillary line (fourth ICS-AAL), or the fifth intercostal space at the midaxillary line (fifth ICS-MAL) for tension pneumothorax in a prehospital environment. Although The 5 cm thoracentesis needle was widely used in clinical practice [11], the failure rate of needle thoracostomy for tension pneumothorax is considerably high, ranging from 4% to 65% [12]. Inadequate needle length less than chest wall thickness (CWT) has been identified as the main cause of failed decompression in many studies [13–15]. It has been controversial in recent years as to which position is most ideal for decompression in general and also in particular to age and ethnicities; some of the relevant studies and their findings are showed in Table 1. In 2018, according to newly issued the Advanced Trauma Life Support (ATLS) guidelines, the fifth ICS-MAL was suggested as the preferred place, and an 8 cm needle rather than the common 5 cm needle was proved to increase success rate of adults' decompression [16]. Nevertheless, no specific recommendations are made for older patients. Older patients are undergoing significant changes in their muscles, hearts, and lungs, thus the CWT may differ from ages. However, literature about the effect of age on the CWT in older patients is rare, and the appropriateness of an 8 cm needle lacks evidence.

Therefore, this study aims to compare two insertion points: the second ICS-MCL and the fifth ICS-MAL and to evaluate the ideal length of the thoracentesis needle based on the success rate and risk of severe complications. Besides, this study explored the influence of age, sex, presence or absence of chronic obstructive pulmonary disease (COPD), and body mass index (BMI) on the CWT at different intercostal spaces to estimate the CWT and select the appropriate length of the thoracentesis needle.

## 2. Materials and Methods

The study was authorized and approved by the Ethics Committee of Chinese PLA General Hospital (2022-041), and the requirement for consent was waived, as this was a retrospective study. This retrospective observational study included consecutive older patients in the Chinese PLA General Hospital, Beijing, from July 1 to 31, 2020. Patients aged ≥ 75 years and in-patients who underwent chest computed tomography (CT) were brought into the study. Patients with a history of chest surgery and patients whose arms were not raised above their heads in their chest CT images were excluded from the study. Clinical data, including patient age, sex, weight, height, and presence or absence of COPD, and imaging data were extracted from electronic medical document.

### 2.1. CWT Measurement

**Figure 1:**
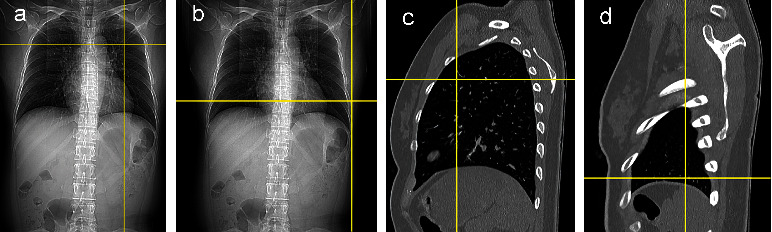
Different insertion points in different positions. Insertion points of the second ICS-MCL (a) and fifth ICS-MAL (b) on a coronal scout topogram. Corresponding insertion point of the second ICS-MCL (c) and fifth ICS-MAL (d) on the sagittal multiplanar reformatted image. Second ICS-MCL, second intercostal space at the midclavicular line, fifth intercostal space at the mid-axillary line.

CWT is the distance from the skin to the parietal pleura. Chest CT was performed on Optima CT660 (GE Medical Systems, Forchheim, Germany), which is a 128-detector scanner with tube voltage 120.0 kV and nominal single collimation width of 1.25 mm. First, a line was drawn along the clavicle on the coronal scout topogram, and the midpoint was marked. Then, a vertical line bisecting the midpoint was dropped into the hemithorax to mimic the clinical determination and to estimate the midclavicular line. The intersection point of the midclavicular line and the horizontal line crossing the inferior border of the second intercostal space (ICS) was considered as the insertion point at the second ICS-MCL (Figure 1(a)). Similarly, the midaxillary line was defined as the vertical line crossing the center of the armpit. The intersection point of the midaxillary line and the horizontal line crossing the inferior border of the fifth ICS was considered as the insertion point at the fifth ICS-MAL (Figure 1(b)). The cross-sectional slices obtained from CT were reconstructed into 5 mm-thick sagittal multiplanar reformatted images. The corresponding insertion points at the second ICS-MCL and fifth ICS-MAL are shown in Figures 1(c) and 1(d). Considering that a longer needle will increase the incidence of severe complications, we assessed the safety of thoracentesis with the shortest depth to vital structure (DVSclose). The DVSclose was the minimum distance from the skin to the vital intrapleural structures crossing the insertion point [11, 17, 18]; it is an index for measuring the safety of needle decompression. These vital structures included the pericardium, aorta, superior vena cava, inferior vena cava, large pulmonary vessels, and thymus gland [17].

**Figure 2:**
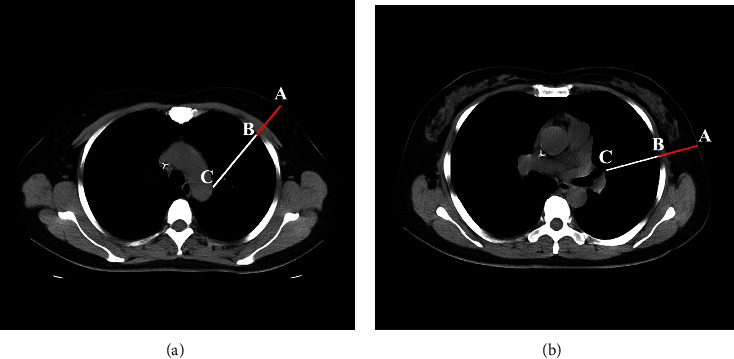
Measurements in different thoracentesis site. Measurements of the radial depth (the segment AB), and DVSclose (the segment AC) in the right second ICSMCL (a) and fifth ICS-MAL (b) second ICS-MCL, second intercostal space at the midclavicular line; fifth ICS-MAL, fifth intercostal space at the mid-axillary line; DVSclose, smallest depth to vital structure.

The radial depth, the shortest depth from the skin to the parietal pleura, was used as representative of the CWT in our study because the CWT varies when the insertion angle change. Measurements were made for each ICS: the radial depth and DVSclose of the second ICS-MCL and fifth ICS-MAL; the measurements were conducted on the left and right sides. Examples of these measurements are shown in Figures 2(a) and 2(b), respectively. In Figure 2, the segment AB denote the CWT and the segment AC denote the DVSclose of the insertion point.

The thoracentesis was considered unsuccessful when the CWT exceeded the needle length. The expected failure rate was calculated using the following equation: expected failure rate = (the number of measurements of CWT of the ICS that exceeded needle length/the number of overall measurements) × 100%. Severe complication should be considered when the DVSclose is less than the length of the needle. The expected severe complication rate was calculated using the following equation: expected severe complication rate = (the number of measurements of DVSclose of the ICS that was less than needle length/the number of overall measurements) × 100%.

### 2.2. Clinical Data Analysis

IBM SPSS Statistics for Windows, version 22.0 (SPSS Inc., Chicago, IL, USA) was used for all statistical analyses. The paired-sample *t*-test was used to compare the CWT and DVSclose between the second ICS-MCL and the fifth ICS-MAL in the same patient. Fisher's exact test was used to compare the expected failure rate and the incidence of severe complications. Then, multiple linear regression was used to analyze the influence between age, sex, presence or absence of COPD, and BMI on the CWT of the second ICS-MCL and the fifth ICS-MAL.

## 3. Results

A total of 91 men and 45 women were finally included in the study. The average age of the patients was 84.9 ± 5.81 years, with a mean BMI of 23.22 ± 3.96 kg/m^2^. The CWT, DVSclose, and difference of them between the second ICS-MCL and the fifth ICS-MAL are shown in Table 2. The results of the paired-sample *t*-test showed that the CWT and DVSclose at the second ICS-MCL was both significantly less than that at the fifth ICS-MAL on both sides of the chest (*P* < 0.05).

Tables 3 and 4, respectively, shows the differences in the expected failure rate and the incidence of severe complications associated with 5 cm versus 7 cm needles and 7 cm versus 8 cm needles. The results showed that success rate associated with using a 7 cm needle was significantly higher than that with using a 5 cm needle (*P* < 0.05), and the incidence of severe complications associated with using a 7 cm needle was significantly less than that with using an 8 cm needle (*P* < 0.05). However, the success rate of the 7 cm and 8 cm needles had no significant difference.

Multiple linear regression analysis showed that the CWT of the second ICS-MCL was significantly correlated with age, sex, presence or absence of COPD, and BMI (*P* < 0.05), whereas the CWT of the fifth ICS- MAL was significantly correlated with sex and BMI (*P* < 0.05). Coefficient of the multiple linear regression (*β*) of different independent variables for different insertion points are shown in Table 5.

## 4. Discussion

As there were few studies discussing the appropriate location and needle length of pneumothorax decompression in elder, it is important to evaluate the distribution of the CWT and DVSclose in older patients to improve the success rate of prehospital pneumothorax decompression and reduce insertion-related complications. This study compared the second ICS-MCL and the fifth ICS-MAL to determine a more appropriate insertion point and described the ideal thoracentesis needle length. In addition, the affecting factors of CWT were also analyzed.

The choice of the insertion points and needle length was a widely studied question but remains controversial so far. In a study by Inaba et al. involving 20 cadavers [12], the success rate of using a 5 cm needle at the fifth ICS was 100%, whereas that at the second ICS was only 58%, indicating that the CWT at the second ICS was relatively thicker. A meta-analysis by Laan et al. indicated that the CWT at the fourth or fifth ICS-AAL was smaller than that at the second ICS-MCL in multiple populations [19]. Elhariri et al. pointed out that the CWT at the fifth ICS-MAL was significantly less than second ICS-MCL and an 8 cm length catheter had a better efficacy in comparison to 5 cm catheter [16]. Previous studies have mainly focused on adults, and there are only few studies on the CWT of the older population. Whether the recommendations for young adults are applicable to the older patients is debatable. Therefore, this study discussed the problem for the older patients and further explored the affecting factors of the CWT.

Given tissue can be displaced by the pressure from the ultrasound probe, altering the CWT and leading to lower measurements than the actual values, CT was used as measurement method instead of ultrasound [20]. Serious complications, such as aortic injury, myocardial injury, and pericardial tamponade, were more likely to occur when using longer thoracentesis needles. Therefore, DVSclose was used as an indicator for the safety of thoracentesis needles in the study.

The 2018 ATLS guidelines recommend the fifth ICS-MAL as the primary location for decompression of tension pneumothorax [16]. However, our research found that the CWT of the second ICS-MCL was significantly less than that of the fifth ICS-MAL both on the left and the right side. Consequently, the second ICS-MCL was associated with a higher success rate than the fifth ICS-MAL for older patients. In addition, according to the common sense, thoracentesis at the fifth ICS-MAL increase the difficulty in transport of the patients and may increase the risk of catheter slippage. The second ICS-MCL may be the better insertion point for older patients, with a thinner CWT and higher success rate.

Regarding needle length, several CT-based studies [20–23] have reported the inadequacy of a common used 5 cm thoracentesis needle for successful decompression. A meta-analysis by Clemency et al. showed that a 6.44 cm thoracentesis needle was needed for a 95% success rate and an 8 cm thoracentesis needle was needed for a 100% success rate at the second ICS-MCL [15]. Yamagiwa et al. pointed out that the CWT varies from nationalities or races [21]. The average CWT at the second ICS-MCL in Japan is 3.06 cm, thus a 5 cm needle is appropriate for 94% of the patients. This study finds that a lower expected failure rate of thoracentesis is associated with using a 7 cm needle compared with a 5 cm needle (*P* < 0.05). Meanwhile, a lower rate of severe complications is associated with a 7 cm needle than an 8 cm needle (*P* < 0.05). The 5 cm thoracentesis needle can't achieve satisfactory goals of success depression but increased the risk of aerodermectasia, thus it was not recommended by the study. In addition, using the 7 cm thoracentesis needle reduced the probability of serious complications but did not significantly decrease the expected failure rate when compared with an 8 cm needle. Therefore, the 7 cm thoracentesis needle is recommended for prehospital decompression of tension pneumothorax for the older Chinese patients.

As for the affecting factors of CWT, many studies have shown that the chest wall of women is thicker than that of men at the second ICS-MCL and fourth or fifth ICS-MAL [13, 21, 22, 24]. Inaba et al. [25] and Powers et al. [26] suggested that there is a relationship between the CWT of second ICS-MCL and BMI, but the relationship between BMI and the fifth ICS-MAL is unknown. The present study showed that CWT of the second ICS-MCL was significantly correlated with age, sex, presence or absence of COPD, and BMI, whereas the CWT of the fifth ICS-MAL was significantly correlated with sex and BMI. It is obvious that patients with higher BMI or female patients, especially with rich subcutaneous tissue of the breasts, are inclined to have thicker CWT, while patients who have COPD tend to have the barrel chest [27], are inclined to have thinner CWT. Almost 20% of elderly can be diagnosed with sarcopenia, which would lead to skeletal muscle loss [28], suggesting that aging leads to a loss of chest wall muscle in older adults [29]. Due to the loss of muscles, older patients tend to have smaller CWT. It is noteworthy that the CWT of the fifth ICS-MAL was not significantly corrected with age and COPD. The reason may be attributed to the less muscle in the fifth ICS-MAL, which may be not notably affected by age or COPD. Further researches should take more affecting factors into consideration and propose a predictive model of the CWT, so that accurate estimation can be achieved in the selection of thoracentesis needles.

This study had some limitations. First, this study only selected inpatients for data integrity; thus, the conclusions may have biased outcomes. Second, the measurement of the CWT relies on manual measurement by the imaging system, and the measurement error is difficult to control. Further research involving more population and using more precise measurement methods would be encouraging.

## 5. Conclusions

This study intended to recommend appropriate location and needle length and to explore the affecting factors of the CWT for older patients. Our study found out the CWT of the second ICS-MCL was significantly less in comparison to the fifth ICS-MAL both on the left and the right side. Therefore, we can induce that, different from the adults' primary thoracentesis site, the second ICS-AAL is the primary site for tension pneumothorax in Chinese patients over 75 years old. In addition, a 7 cm thoracentesis needle, with significant decrease in the incidence of severe complications, may be a better option than the 8 cm thoracentesis needle for Chinese patients over 75 years old. Doctors should mainly consider age, sex, presence or absence of COPD, and BMI when choosing the proper needle length.

## Figures and Tables

**Table 1:** Summary of recommended positions of needle decompression and catheter sizes in different studies.

Authors	Nation	Method	No. of patients (male)	Suggestion on thoracentesis site	Suggestion on catheter size
Yamagiwa et al. [21]	Kanagawa, Japan	CT	256 (192)	None	>94% of Japanese trauma patients can be treated with a 5 cm catheter
Akoglu et al. [24]	Zonguldak, Turkey	CT	160 (136)	The fifth ICS-MAL is a better option for a puncture site	5 cm catheter is unlikely to access pleural space in at least one-third of female and one-tenth of male Turkish trauma patients
Inaba et al. [12]	California, USA	Thoracotomy	20 (14)	CWT was 1 cm less at the fifth ICS-MAL vs the second ICS-MCL on average, the fifth ICS-MAL is a better site	100% of needles placed in the fifth ICS-MAL and 57.5% in the second ICS-MCL entered chest cavity with a 5 cm needle
Harcke et al. [11]	Washington, USA	CT	101 (101)	None	An 8 cm catheter would have reached the pleural space in 99% of the patients
Nelson et al. [30]	New York, USA	Ultrasound	30 (19)	The patients have a smaller chest wall distance at the fifth ICS-MAL vs. the second ICS-MCL. The fifth ICS was supported as a better site	None
Chang et al. [31]	North Carolina, USA	CT	100 (84)	CWT at the fourth ICS-AAL is significantly thinner than the second ICS-MCL	8 cm catheter have higher chance of pleural decompression when compared with 5 cm catheters
Powers et al. [26]	North Carolina, USA	CT	326 (228)	None	On average, patients needed catheter length of 6.0–6.5 cm to successfully decompression

CT, computerized tomography; fifth ICS-MAL, fifth intercostal space at the midaxillary line; second ICS-MCL, the second intercostal space at the midclavicular line; fourth ICS-AAL, fourth intercostal space at the anterior axillary line.

**Table 2:** Comparison of CWT between the second ICS-MCL and fifth ICS-MAL on different planes.

	Radial depth		DVSclose	
	Left	Right	Left	Right
Second ICS-MCL	3.22 (1.03)	3.18 (1.02)	8.99 (1.53)	8.85 (1.24)
Fifth ICS-MAL	3.65 (1.51)	3.91 (1.72)	9.83 (1.96)	10.40 (2.16)
95% CI	(−0.61, −0.25)	(−0.94, −0.53)	(−1.01, −0.61)	(−1.91, −1.20)
*P*	0.001	0.001	0.001	0.001

Data are expressed as mean ± standard deviation. *P* values of the paired-samples *t*-test are shown in the table. CWT, chest wall thickness; second ICS-MCL, second intercostal space at the midclavicular line; fifth ICS-MAL, fifth intercostal space at the midaxillary line.

**Table 3:** Comparison of the expected failure rate and the incidence of severe complications with different needle lengths.

Needle lengths	Number of failures (n/all)		Number of severe complications (*n*/all)	
	second ICS-MCL	fifth ICS-MAL	second ICS-MCL	fifth ICS-MAL
5 cm	10/272	48/272	2/272	5/272
7 cm	1/272	13/272	15/272	10/272
*P*	0.011	0.001	0.002	0.295

*P* values of the Fisher's exact test are shown in the table. Second ICS-MCL, second intercostal space at the midclavicular line; fifth ICS-MAL, fifth intercostal space at the midaxillary line.

**Table 4:** Comparison of the expected failure rate and the incidence of severe complications with different needle lengths.

Needle lengths	Number of failures (n/all)		Number of severe complications (*n*/all)	
	second ICS-MCL	fifth ICS-MAL	second ICS-MCL	fifth ICS-MAL
7 cm	1/272	13/272	15/272	10/272
8 cm	0/272	6/272	59/272	31/272
*P*	1.000	0.254	0.001	0.001

*P* values of the Fisher's exact test are shown in the table. second ICS-MCL, second intercostal space at the midclavicular line; fifth ICS-MAL, fifth intercostal space at the midaxillary line.

**Table 5:** Coefficient of the multiple linear regression (*β*) of different independent variables for different insertion points.

Insertion points	Sex	Age	BMI	COPD
Left second ICS-MCL	−0.21	−0.15	0.76	−0.19
Right second ICS-MCL	−0.24	−0.16	0.82	−0.17
Left fifth ICS-MAL	−0.36	NA	0.73	NA
Right fifth ICS-MAL	−0.34	NA	0.72	NA

Second ICS-MCL, second intercostal space at the midclavicular line; fifth ICS-MAL, fifth intercostal space at the midaxillary line; BMI, body mass index; COPD, obstructive pulmonary disease; NA: the factor has no significant influence on the dependent variable in the multiple linear regression.

## Data Availability

The datasets generated during and/or analyzed during the current study are available from the corresponding author on reasonable request.

## References

[1] quality-programs (2016). National trauma data bank 2016 annual report. https://www.facs.org/-/media/files/quality-programs/trauma/ntdb/ntdb-annual-report-2016.ashx.

[2] Demetriades D., Sava J., Alo K. (2001). Old age as a criterion for trauma team activation. *The Journal of Trauma, Injury, Infection, and Critical Care*.

[3] Benjamin E. R., Khor D., Cho J., Biswas S., Inaba K., Demetriades D. (2018). The age of undertriage: current trauma triage criteria underestimate the role of age and comorbidities in early mortality. *Journal of Emergency Medicine*.

[4] Brooks S. E., Mukherjee K., Gunter O. L. (2014). Do models incorporating comorbidities outperform those incorporating vital signs and injury pattern for predicting mortality in geriatric trauma?. *Journal of the American College of Surgeons*.

[5] Ahl R., Phelan H. A., Dogan S., Cao Y., Cook A. C., Mohseni S. (2017). Predicting in-hospital and 1-year mortality in geriatric trauma patients using geriatric trauma outcome score. *Journal of the American College of Surgeons*.

[6] Hurewitz A. N., Sidhu U., Bergofsky E. H. (1986). Cardiovascular and respiratory consequences of tension pneumothorax. *Bulletin Europeen de Physiopathologie Respiratoire*.

[7] Warner K. J., Copass M. K., Bulger E. M. (2008). Paramedic use of needle thoracostomy in the prehospital environment. *Prehospital Emergency Care*.

[8] Galvagno S. M., Nahmias J. T., Young D. A. (2019). Advanced trauma life Support(®) update 2019: management and applications for adults and special populations. *Anesthesiology Clinics*.

[9] Kortbeek J. B., Al Turki S. A., Ali J. (2008). Advanced trauma life support, 8th edition, the evidence for change. *The Journal of Trauma, Injury, Infection, and Critical Care*.

[10] Chapleau W. (2001). PHTLS (prehospital trauma life support) overseas. *Emergency Medical Services*.

[11] Harcke H. T., Pearse L. A., Levy A. D., Getz J. M., Robinson S. R. (2007). Chest wall thickness in military personnel: implications for needle thoracentesis in tension pneumothorax. *Military Medicine*.

[12] Inaba K., Branco B. C., Eckstein M. (2011). Optimal positioning for emergent needle thoracostomy: a cadaver-based study. *The Journal of Trauma, Injury, Infection, and Critical Care*.

[13] Givens M. L., Ayotte K., Manifold C. (2004). Needle thoracostomy: implications of computed tomography chest wall thickness. *Academic Emergency Medicine*.

[14] Schroeder E., Valdez C., Krauthamer A. (2013). Average chest wall thickness at two anatomic locations in trauma patients. *Injury*.

[15] Clemency B. M., Tanski C. T., Rosenberg M., May P. R., Consiglio J. D., Lindstrom H. A. (2015). Sufficient catheter length for pneumothorax needle decompression: a meta-analysis. *Prehospital and Disaster Medicine*.

[16] Yehia Elhariri S., Mohamed H., As Burud I., Elhariri A. (2019). Changing trends in the decompression of tension pneumothorax. *Journal of Surgery and Research*.

[17] Terboven T., Leonhard G., Wessel L. (2019). Chest wall thickness and depth to vital structures in paediatric patients - implications for prehospital needle decompression of tension pneumothorax. *Scandinavian Journal of Trauma, Resuscitation and Emergency Medicine*.

[18] Mandt M. J., Hayes K., Severyn F., Adelgais K. (2019). Appropriate needle length for emergent pediatric needle thoracostomy utilizing computed tomography. *Prehospital Emergency Care*.

[19] Laan D. V., Vu T. D., Thiels C. A. (2016). Chest wall thickness and decompression failure: a systematic review and meta-analysis comparing anatomic locations in needle thoracostomy. *Injury*.

[20] Sanchez L. D., Straszewski S., Saghir A. (2011). Anterior versus lateral needle decompression of tension pneumothorax: comparison by computed tomography chest wall measurement. *Academic Emergency Medicine*.

[21] Yamagiwa T., Morita S., Yamamoto R., Seki T., Sugimoto K., Inokuchi S. (2012). Determination of the appropriate catheter length for needle thoracostomy by using computed tomography scans of trauma patients in Japan. *Injury*.

[22] Zengerink I., Brink P. R., Laupland K. B., Raber E. L., Zygun D., Kortbeek J. B. (2008). Needle thoracostomy in the treatment of a tension pneumothorax in trauma patients: what size needle?. *The Journal of Trauma, Injury, Infection, and Critical Care*.

[23] Drinhaus H., Annecke T., Hinkelbein J. (2016). Chest decompression in emergency medicine and intensive care. *Anaesthesist, Der*.

[24] Akoglu H., Akoglu E. U., Evman S. (2013). Determination of the appropriate catheter length and place for needle thoracostomy by using computed tomography scans of pneumothorax patients. *Injury*.

[25] Inaba K., Ives C., McClure K. (2012). Radiologic evaluation of alternative sites for needle decompression of tension pneumothorax. *Archives of Surgery*.

[26] Powers W. F., Clancy T. V., Adams A., West T. C., Kotwall C. A., Hope W. W. (2014). Proper catheter selection for needle thoracostomy: a height and weight-based criteria. *Injury*.

[27] Sarkar M., Bhardwaz R., Madabhavi I., Modi M. (2019). Physical signs in patients with chronic obstructive pulmonary disease. *Lung India*.

[28] Dao T., Green A. E., Kim Y. A. (2020). Sarcopenia and muscle aging: a brief overview. *Endocrinology and metabolism (Seoul, Korea)*.

[29] Skloot G. S. (2017). The effects of aging on lung structure and function. *Clinics in Geriatric Medicine*.

[30] Nelson M., Chavda Y., Stankard B., McCann-Pineo M., Nello A., Jersey A. (2022). Using ultrasound to determine optimal location for needle decompression of tension pneumothorax: a pilot study. *Journal of Emergency Medicine*.

[31] Chang S. J., Ross S. W., Kiefer D. J. (2014). Evaluation of 8.0-cm needle at the fourth anterior axillary line for needle chest decompression of tension pneumothorax. *Journal of Trauma and Acute Care Surgery*.

